# IMPLEMENTING VISUAL VIDEOS AND IMAGES WITH PEOPLE WITH DEMENTIA IN CARE SETTINGS: A SCOPING REVIEW

**DOI:** 10.1093/geroni/igac059.2805

**Published:** 2022-12-20

**Authors:** Karen Lok, Yi Wong, Mario Bayani, Jim Mann, Annette Berndt, Lily Wong, Carly Wang, Diane Pan, Lillian Hung

**Affiliations:** University of British Columbia, Vancouver, British Columbia, Canada; University of British Columbia, Vancouver, British Columbia, Canada; Community Engagement Advisory Network, Vancouver, British Columbia, Canada; Community Engagement Advisory Network, Vancouver, British Columbia, Canada; Community Engagement Advisory Network, Vancouver, British Columbia, Canada; Community Engagement Advisory Network, Vancouver, British Columbia, Canada; University of British Columbia, Vancouver, British Columbia, Canada; University of British Columbia, Vancouver, British Columbia, Canada; University of British Columbia, Vancouver, British Columbia, Canada

## Abstract

There is limited literature on using visual videos and images with people with dementia in care settings. We conducted a scoping review on this topic to fill this literature gap. Our scoping review adopted the Joanna Briggs Institute scoping review methodology. We eventually included eleven papers for the review and conducted the content analysis. We found the facilitators for implementing visual videos and images with people with dementia in care settings: 1. Matching people’s interests 2. Being congruent with people’s cognitive abilities 3. Support from families and staff 4. Using in a group setting. We also found the barriers: 1. Staff is unwilling to support 2. Lack of resources 3. Not congruent with the cognitive or other abilities of the people. We found benefits of using visual videos and images with this population: 1. Encourage expression 2. Facilitate discussions with other people 3. Improve well-being. We also found drawbacks: the potential of arousing negative emotions and memories. We suggest future research should include the voices of people with dementia, staff should be trained to support the people in case negative memories and emotions are aroused, and there should be consideration of using visual videos and images to tackle isolation and loneliness in care settings. With these findings, this scoping review should shed light on implementing visual videos and images in care settings.

